# Impact of Mental Health and Substance Use Disorders on Emergency Department Visit Outcomes for HIV Patients

**DOI:** 10.5811/westjem.2016.1.28310

**Published:** 2016-03-02

**Authors:** Brian Y. Choi, Diana M. DiNitto, C. Nathan Marti, Namkee G. Choi

**Affiliations:** * Warren Alpert Medical School at Brown Univeristy and Rhode Island Hospital, Department of Emergency Medicine, Providence, Rhode Island; † The University of Texas at Austin, School of Social Work, Austin, Texas

## Abstract

**Introduction:**

A disproportionate number of individuals with human immunodeficiency virus (HIV) have mental health and substance-use disorders (MHSUDs), and MHSUDs are significantly associated with their emergency department (ED) visits. With an increasing share of older adults among HIV patients, this study investigated the associations of MHSUDs with ED outcomes of HIV patients in four age groups: 21–34, 35–49, 50–64, and 65+ years.

**Methods:**

We used the 2012 Nationwide Emergency Department Sample (NEDS) dataset (unweighted n=23,244,819 ED events by patients aged 21+, including 115,656 visits by patients with HIV). Multinomial and binary logistic regression analyses, with “treat-and-release” as the base outcome, were used to examine associations between ED outcomes and MHSUDs among visits that included a HIV diagnosis in each age group.

**Results:**

Mood and “other” mental disorders had small effects on ED-to-hospital admissions, as opposed to treat-and-release, in age groups younger than 65+ years, while suicide attempts had medium effects (RRR=3.56, CI [2.69–4.70]; RRR=4.44, CI [3.72–5.30]; and RRR=5.64, CI [4.38–7.26] in the 21–34, 35–49, and 50–64 age groups, respectively). Cognitive disorders had medium-to-large effects on hospital admissions in all age groups and large effects on death in the 35–49 (RRR=7.29, CI [3.90–13.62]) and 50–64 (RRR=5.38, CI [3.39–8.55]) age groups. Alcohol use disorders (AUDs) had small effects on hospital admission in all age groups (RRR=2.35, 95% CI [1.92–2.87]; RRR=2.15, 95% CI [1.95–2.37]; RRR=1.92, 95% CI [1.73–2.12]; and OR=1.93, 95% CI [1.20–3.10] in the 21–34, 35–49, 50–64, and 65+ age groups, respectively). Drug use disorders (DUDs) had small-to-medium effects on hospital admission (RRR=4.40, 95% CI [3.87–5.0]; RRR=4.07, 95% CI [3.77–4.40]; RRR=4.17, 95% CI [3.83–4.55]; and OR=2.53, 95% CI [2.70–3.78] in the 21–34, 35–49, 50–64, and 65+ age groups, respectively). AUDs and DUDs were also significantly related to the risk of death, and DUDs had a small effect on the risk of discharge against medical advice in the 35–49 and 50–64 age groups.

**Conclusion:**

The high prevalence of MHSUDs and their significant roles in ED visit outcomes in patients with HIV provide support for integrated care for these patients outside the ED to reduce their ED visits and costly hospital admissions and institutional care that follows, especially for the increasing numbers of older adults with HIV.

## INTRODUCTION

Hospital emergency departments (ED) are one of the most frequent sources of medical care for many individuals with human immunodeficiency virus (HIV), and ED patients with HIV use significantly more ED resources than patients without HIV.^1^,^2^ A study based on the National Hospital Ambulatory Medical Care Survey (NHAMCS) estimated that persons with HIV made about five in 1,000 ED visits, with the highest visit rates found among those aged 45–54, Blacks, those with public medical insurance, and residents of metropolitan areas.^3^ Compared to patients without HIV, those with HIV also had a longer duration of ED stays (5.4 hours vs. 3.6 hours) and were more likely to be admitted to a hospital (28% vs. 15%) despite no recorded difference in the acuity level of the two groups’ presenting problem(s).^3^

Previous research shows that a disproportionate number of individuals with HIV have mental health and substance-use disorders (MHSUDs) and that ED visits by persons with HIV are significantly associated with MHSUDs.^4^–^6^ The most common mental health problems in persons living with HIV are major depression (20–35%), anxiety disorders (19–37%), post-traumatic stress disorders (PTSD) (15–26%), and severe mental illnesses (5–23%; schizophrenia, schizoaffective disorder, bipolar disorder, and other Axis 1 disorders).^6^,^7^ Substance (alcohol and/or drug) use disorders were also found among 7–16% of persons living with HIV.^6^,^7^ MHSUDs have been linked to delayed access and non-adherence to highly active antiretroviral therapy (HAART) or combination antiviral therapy (cART), treatment dropout, and worse disease outcomes; and higher symptom severities were associated with lower HAART adherence rates.^6^–^10^ Depression may be linked to non-adherence to treatment through low motivation to seek care, loss of interest in continuing with care, and feelings of hopelessness about the future; anxiety and PTSD may negatively affect treatment because they may hamper concentration; and SUDs are likely to negatively affect treatment because they impair memory, concentration, impulse control, and the patient-provider relationship.^6^ Health crises stemming from MHSUDs or from MHSUD-influenced delays in receiving HIV treatment, poor treatment adherence, and treatment cessation are likely to increase the need for ED visits. Compared to the general population, individuals with HIV also have higher (3+ times) rates of suicidal ideation and attempts, and ideators and attempters with HIV have higher rates of MHSUDs.^11^,^12^

Of individuals who have HIV, those with MHSUDs tend to have lower socioeconomic status and more complex care needs for HIV and other comorbid medical issues; as a result, they have increased healthcare utilization and costs of care compared to HIV patients in general, although access to care among those with MHSUDs is often suboptimal.^13^,^14^ Life expectancy of individuals with HIV has increased due to HAART. Persons aged 55+ years accounted for 26% (313,200) of the estimated 1.2 million people living with HIV infection in the United States in 2011, and there were an estimated 8,575 new HIV diagnoses among people aged 50+, with 44% (n=3,747) of them among those aged 50–54.^15^ MHSUDs may further complicate care and affect care outcomes of older individuals with HIV because they are more likely to have other chronic illnesses, including metabolic dysregulation, cardiovascular disease, and chronic pulmonary disease, than younger people with HIV.^16^,^17^

Research on ED visits by people with HIV and MHSUDs has generally not considered potential age group difference. The present study investigated ED outcomes among four groups of adults with HIV, with and without a diagnosis of MHSUD: those aged 21–34 years; 35–49 years; 50–64 years, and 65+ years. Our hypotheses were that (1) ED visits by older (aged 50–64 and 65+ years) than younger adults with HIV will be more likely to result in hospital admission and/or other outcomes than treat-and-release; and (2) HIV patients with MHSUDs in each age group will be more likely to result in hospital admission or other outcomes than treat-and-release.

## METHODS

### Data and Sample

Data came from the 2012 Nationwide Emergency Department Sample (NEDS) sponsored by the Agency for Healthcare Research and Quality. This publicly available dataset is part of the Healthcare Cost and Utilization Project and is the largest all-payer ED database. In 2012 NEDS contained information on 31 million ED visits at 950 hospitals in 30 states and approximated a 20% stratified sample of all hospital-based EDs in the United States.^18^ Stratification was based on geographic region, trauma center designation, hospitals’ urban or rural location, teaching hospitals, and hospital ownership/control (public, for-profit, and not-for-profit). The 31 million ED events contained in the 2012 NEDS are weighted to represent the estimated 134 million ED events nationwide in that year.^18^ In this study, we focused on the 23,244,819 ED events by patients aged 21+ (representing 100,329,568 weighted events or 74.7% of all 134 million ED visits by all age groups). We excluded the under-21 age group as HIV diagnosis was found in just 0.02% (n=1,677) of all visits by this age group. Of the 23,244,819 ED events, 115,656 events (representing 489,285 weighted events) were by persons with HIV.

NEDS data elements include patient demographics (age and gender); patient location (in counties by population size); patient zip code area income (in national quartiles); diagnostic and procedure codes from the International Classification of Diseases, 9^th^ Revision, Clinical Modification (ICD-9-CM), as well as the clusters/categories of diagnoses in the Clinical Classifications Software (CCS) system;^19^ chronic condition indicator (ICD-9-CM diagnoses that last 12 months or longer and place limitations on self-care/independent living/social interactions and/or result in the need for ongoing intervention with medical products/services/special equipment); E Codes (for external causes of injury and poisoning: self-inflicted, intentional; unintentional; and assault-related); total charges; and ED dispositions/outcomes.

### Measures

HIV diagnosis was identified from the single-level CCS diagnosis (i.e., HIV infection) classification as long as it was listed as a diagnosis, ranging from the primary to the fifteenth diagnosis. Of the visits with HIV diagnosis, it was the 1^st^ diagnosis for 9.9%; 2^nd^ for 31.0%; 3^rd^ for 20.6%; 4^th^ for 12.8%; 5^th^ for 8.4%; 6^th^ for 5.5%; 7^th^ for 3.5%; 8^th^ for 2.5%; 9^th^ for 1.7%; 10^th^ for 1.2%; 11^th^ for 0.8%; 12^th^ for 0.7%; 13^th^ for 0.5%; 14^th^ for 0.3%; and 15^th^ for 0.6%.

MHSUDs were identified from the single-level CCS diagnosis classifications including 12 mental health disorders (suicide is one of them) and two substance use disorders—alcohol use disorders (AUDs) and drug use disorders (DUDs)—as long as they were listed as a diagnosis (ranging from the primary to the fifteenth diagnosis). We further collapsed the 12 mental health disorders into five: (1) anxiety disorders; (2) mood disorders; (3) delirium/dementia, amnestic, and other cognitive disorders (collectively referred to as cognitive disorders hereafter); (4) other mental health disorders (adjustment disorders; attention deficit, conduct, and disruptive behavior disorders; developmental disorders; impulse control disorders; personality disorders; schizophrenia and other psychosis; and other miscellaneous disorders); and (5) suicide or suicide attempt.

ED outcomes were (1) treat-and-release; (2) admission to the same hospital or transfer to a short-term hospital as an inpatient (and did not die); (3) death either in the ED or in the hospital; (4) transfer to a skilled nursing facility, intermediate care facility, or other such facility (transfer to another facility); or discharge with initiation of home healthcare services (HHC); (5) discharge against medical advice (AMA); and (6) other/discharge with an unknown destination. Transfer to another facility and HHC were combined because of small sample size in each category.

Potential confounders included: (1) gender (2) patient zip code area income (lower 50%, missing, and upper 50% [reference category]); (3) the following 11 clusters of diagnosed physical illnesses, in addition to infectious (since all patients with HIV have this condition) and parasitic diseases, based on the single-level CCS diagnosis classifications (yes=1, no=0 for each): a) neoplasms; b) endocrine, nutritional, and metabolic diseases and immunity disorders; c) diseases of blood and blood-forming organs; d) diseases of the nervous system and sense organs; e) diseases of the circulatory system; f) diseases of the respiratory system; g) diseases of the digestive system; h) diseases of the genitourinary system; i) diseases of the skin and subcutaneous tissues; j) diseases of the musculoskeletal system and connective tissue; and k) other conditions; and (4) any non-suicidal external injury from the E codes.

### Data Analysis

We conducted analyses with Stata/MP 14’s svy function to account for NEDS’s multi-stage, stratified sampling design. Stata’s subpop command was used for all subsample analyses of visits by individuals aged 21+ to ensure that variance estimates incorporate the full sampling design. All estimates presented are weighted to discharges in the universe except for sample sizes (i.e., number of visits). Standard errors for all study variables show stable estimates. First, we ued χ^2^ tests to describe patient characteristics first by HIV status (no diagnosis vs. diagnosis) and second by age group among patients with HIV. Second, multinomial logistic regression analyses were used to examine associations between ED outcomes (the dependent variable, with treat-and-release as the base outcome) and age group and MHSUDs among all visits that included a HIV diagnosis, with the potential confounders described above. We excluded the ED outcome of “other/unknown” (n=78 for all visits with HIV diagnosis) due to its small sample size. Third, age-group separate multinomial logistic regression analyses were used to examine associations between MHSUDs and ED outcomes within each of the 21–34, 35–49, and 50–64 age groups. The ED outcome of death (n=127) was excluded for the 21–34 age group as the number of covariates exceeded the recommended guideline for the sample exhibiting these outcomes to degrees of freedom ratio (10:1).^20^ Fourth, for the 65+ age group, we used binary logistic regression analysis with ED outcomes of hospital admission versus treat-and-release because the numbers of ED events with other outcomes in this age group were small (n=121 for death; n=95 for transfer/home healthcare; and n=60 for discharge AMA) and resulted in model overfitting. We did not include an interaction term between MH and SUD diagnoses (i.e., any MH disorder and AUD and/or DUD) in final regression models as the interaction effect was not statistically significant (i.e., p>0.20) in our preliminary analysis.

Variance inflation factor diagnostics (using a cut-off of 2.50),^21^ indicated that multicollinearity among the predictors was not a concern. Given the overall large sample size, we followed Chen, Cohen, and Chen’s cutoffs for odds ratios (OR): OR<0.60 or OR=1.68 as equivalent to cutoffs for a small effect size (Cohen’s d=0.2) and OR=3.47 and OR=6.71 as equivalent to cutoffs for medium (d=0.5) and large (d=0.8) effect sizes, respectively.^22^

## RESULTS

### Patient Characteristics by HIV Diagnosis Status

Table 1 shows that 115,656 of the 23 million ED visits by patients 21+ years old, or 0.49% (95% confidence interval=0.48–0.49), had a recorded diagnosis of HIV infection (0.30% of all visits in the 21–34 age group, 0.83% in the 35–49 age group, 0.78% in the 50–64 age group, and 0.09% of in the 65+ age group). Of the visits by patients with HIV, 18.4% were by the 21–34 age group, 42.7% by the 35–49 age group, 34.6% by the 50–64 age group, and 4.3% by the 65+ age group. Thus, almost 40% of the visits by adults (aged 21+) with HIV were by those aged 50+ years. Of the visits by patients without HIV, 30% were by the 21–34 age group, 25.2% were by the 35–49 age group, 21.7% were by the 50–64 age group, and 23.1% were by the 65+ age group.

Compared to visits by those without HIV, visits by those with HIV included higher proportions of men, those with Medicaid as the primary payer, and residents of lower income areas. Visits by those with HIV also included higher proportions of all physical illnesses, except musculoskeletal system and connective tissue problems and non-suicidal external injuries. Mood (15.6% vs. 7.1%) and other mental disorders (6.2% vs. 2.8%), including a diagnosis of schizophrenia or other psychosis (4.4% vs. 1.48%) were more than twice as high in visits with HIV than those without. Suicide attempt was more than three times higher in visits with HIV (2.4% vs. 0.8%). On the other hand, cognitive disorders were lower among the visits with HIV, most likely due to the lower proportion of visits by those aged 65+ in the HIV group compared to the number of visits by those aged 65+ without HIV. SUD rates were also significantly higher in visits by those with HIV. AUD was more than twice as high (7.5% vs. 3.3%), and DUD was almost seven times higher (13.8% vs. 2.7%).

Table 2 shows that fewer visits by those with HIV than those without HIV were treat-and-release (57.3% vs. 77.3%), while more were ED-to-hospital admission (38% vs. 18.9%), death in the ED or during the related hospital admission (1.1% vs. 0.7%), and discharge AMA (2.3% vs. 1.7%). The rate of transfer to another facility/HHC was nearly identical (1.4% and 1.5%). Data also show that differences between those with and without HIV were significant in each age group, though regardless of HIV status, younger groups had higher treat-and-release rates and older groups had higher ED-to-hospital admission rates.

### Patients with HIV Diagnosis: Characteristics by Age Group

Table 3 shows that visits by the 35–49 age group included a higher proportion of women, and visits by the 65+ age group included a higher proportion of men than visits by the other age groups. Visitors aged 65+ also appear to have come from neighborhoods with slightly higher incomes than the younger groups. As expected, however, visits by the 65+ age group included higher proportions of all physical illnesses except skin and subcutaneous tissue conditions, followed by visits by the 50–64 age group. The 21–34 age group had the highest rate of skin conditions. Visits by those aged 65+ also included the highest rate of cognitive disorders but the lowest rates of other MHSUDs, while visits by those aged 35–49 had the highest MHSUD rates followed by visits by the 50–64 age group.

Additional analyses show that HIV infection as the first, second, or third diagnosis was highest in visits by the 21–34 age group (68.7%) and lowest in the 65+ age group (50.4%). Conversely, HIV infection as the 6^th^ through 15^th^ diagnosis was highest in visits by the 65+ age group (28.7%) and lowest in the 21–34 age group (11.3%); thus, the 65+ age group had other more acute physical health and injury-related problems. Regardless of age group, visits in which HIV was either the primary or the 6^th^ through 15^th^ diagnosis were more likely to result in hospital admission than visits in which HIV was the second through the fifth diagnosis.

### Associations of MHSUDs with ED Outcomes Among All Visits with an HIV Diagnosis

Multinomial logistic regression results in Table 4 show that age group’s effects on ED outcomes of hospital admission, death, and discharge AMA, though statistically significant, did not appear to be clinically meaningful after adjusting for confounding variables. However, compared to visits by those aged 21–34, visits by those aged 65+ were associated with an increased risk of transfer to another facility/HHC (relative risk ratio [RRR]=3.08, 95% confidence interval CI [2.36–4.02]). Mood and other mental disorders had small effects (RRR=1.81, 95% CI [1.72–1.90]), and cognitive disorders (RRR=4.52, 95% CI [3.80–5.37]), suicide attempts (RRR=4.35, 95% CI [3.84–4.94]), and DUD (RRR=4.15, 95% CI [3.94–4.37]) had medium effects on increasing the risk of hospital admission. Cognitive disorders had a medium effect (RRR=5.86, 95% CI [4.20–8.17]) and AUD had a small effect (RRR=1.71, 95% CI [1.33–2.19]) on increasing the risk of death while anxiety disorders were associated with a decreased risk (RRR=0.35, 95% CI [0.22–0.57]) of death. Anxiety, mood, and other mental disorders and DUD had small effects on increasing the risk of transfer to another facility/HHC, and cognitive disorders (RRR=6.18, 95% CI [4.44–8.60]) and suicide attempts (RRR=15.77, 95% CI [13.05–19.07]) had medium and large effects, respectively. Suicide attempts decreased the risk of discharge AMA (RRR=0.40, 95% CI [0.21–0.78]), while DUD had a marginal effect (RRR=1.66, 95% CI [1.45–1.90]) on increasing the risk.

### Associations of MHSUDs with ED Outcomes within Each Age Group

Table 5 shows that within the 21–34, 35–49, and 50–64 age groups, mood and other mental disorders and AUD had small effects; cognitive disorders had medium-to-large effects (e.g., RRR=3.70, CI [2.87–4.77] in the 50–64 age group and RRR=11.11, CI [4.60–26.84] in the 21–34 age group); suicide attempts had medium effects (e.g., RRR=3.56, CI [2.69–4.70] in the 21–34 age group and RRR=4.44, CI [3.72–5.30] in the 35–49 age group); and DUD medium effects (e.g., RRR=4.40, CI [3.87–5.00] in the 21–34 age group and RRR=4.17, CI [3.83–4.55] in the 50–64 age group) on increased risk of hospital admission.

Cognitive disorders had a large effect on death in the 35–49 (RRR=7.29, CI [3.90–13.62]) and 50–64 (RRR=5.38, CI [3.39–8.55]) age groups, and AUD had small effect on death in the 35–49 (RRR=2.15, CI [1.95–2.37]) and 50–64 (RRR=1.71, CI [1.22–2.39]) age groups. Mood and other mental disorders had small-to-medium effects, cognitive disorders had medium to large effects, and suicide attempts had large effects on the risk of transfer to another facility/HHC in the 21–34, 35–49, and 50–64 age groups. In addition, anxiety had a small effect in the 50–64 age group and DUD had small effects in the 35–49 and 50–64 age groups on the risk of transfer to another facility/HHC. DUD had a marginal effect on increased risk of discharge AMA in the 35–49 age group. Cognitive disorders and DUD also had small effects on increased risk of discharge AMA in the 50–64 age group.

Table 5 also shows that in the 65+ age group, cognitive disorders had medium effects (OR=6.09, CI [4.03–9.22]), and AUD and DUD had small effects (OR=1.93, CI [1.20–3.10] for AUD and OR=2.53, CI [1.70–3.78] for DUD) on increased risk of hospital admission.

## DISCUSSION

Given the higher rates of MHSUDs and ED utilization among people living with HIV than those without HIV, it is important to examine associations between MHSUDs and ED outcomes among HIV patients. Also, given increasing numbers of older individuals with HIV, healthcare providers need better understanding of potential age-group difference in the association between MHSUDs and healthcare outcomes. Using a nationally representative sample of ED visits, we examined such association in different age groups of ED patients with HIV.

Consistent with the NHAMCS,^3^ the present study found that about five in 1,000 ED visits by U.S. adults age 21+ included an HIV diagnosis and that ED-to-hospital admission was twice as high among those with HIV diagnosis than those without. The present study also shows that more than 77% of ED visits by individuals with HIV were by those aged 35 to 64 years, compared to 47% of ED visits by individuals without HIV. Those aged 65+ constituted only 4% of all ED visits by adults with HIV in 2012, while they were 23% of all ED visits by adults without HIV. However, the large presence of the 50–64 age group (35%) in ED visits by those with HIV signals an upward trajectory of ED visits by older adults with HIV in the future. In the present study, 43% and 53% of ED visits by the 50–64 and 65+ age groups, respectively, with HIV (compared to 22% and 39% for those without HIV) were admitted to the hospital. With age- and HIV-related health/mental health problems, the growing number of older adults with HIV are likely to require more intensive care than their peers without HIV.

Corroborating previous studies,^6^,^7^,^11^ this study found higher rates of MHSUDs among persons living with HIV compared to persons without HIV. Mood disorders, “other” mental disorders, and AUDs were more than twice as high in ED visits by those with HIV than those without; DUDs were more than five times higher; and co-occurring MHSUDs were nearly four times higher. The suicide attempt rate, which is three times higher among those with than without HIV, is also a serious public health concern.

Multivariate analyses showed that among ED visitors younger than age 65 with HIV, mood and “other” mental disorders had small effects on ED-to-hospital admission, as opposed to treat-and-release, and cognitive disorders and suicide attempt had medium-to-large effects on hospital admission as well as transfer to another facility/HHC. Cognitive disorders also had medium effects on the risk of death in the 35–49 and 50–64 age groups. In the 65+ age group, only cognitive disorders had medium effects on hospital admission. With respect to substance use disorders, both AUDs and DUDs had small-to-medium effects on hospital admission in all age groups. Additionally, AUDs had a small effect, and DUDs had a marginal-to-small effect on the risk of discharge AMA in the 35–49 and 50–64 age groups, suggesting that drug addiction may be a barrier to receiving both HIV treatment^9^ and healthcare in general.

An important and consistent finding is the role of cognitive disorders in increasing the likelihood of hospital admissions and transfer to another facility/HHC in all age groups, with the largest effect in the 21–34 age group, and in death in the 35–49 and 50–64 age groups. It is not clear if cognitive disorders among the younger and middle-aged groups are neurocognitive sequelae from alcohol and illicit drug use (which may have contributed to contracting HIV) and/or HIV-associated neurocognitive impairments (HAND). In the older age groups, cognitive disorders may also be age-related dementia and/or HAND.^23^–^25^ Although cART therapy has significantly reduced HAND, mild cognitive disorders are highly prevalent among HIV-positive people.^26^–^28^ One study also found a high prevalence of neurocognitive dysfunction in Romanian young adults growing up with HIV.^29^ Our findings indicate that cognitive disorders are less likely in younger than in older ED patients with HIV, but they can be more detrimental to health and healthcare outcomes in younger adults.

## LIMITATIONS

Our study has several limitations due to NEDS data constraints. First, data on substance misuse were limited to diagnoses of alcohol and drug use disorders. Adverse effects of substance misuse that do not meet diagnostic criteria may also precipitate ED use among people with HIV given their multiple physical and mental health problems. Future research should examine the effects of at-risk and binge/heavy drinking and drug misuse to provide more insight into their effects. Second, HIV diagnosis may not have been recorded in short treat-and-release visits for acute problems that may be unrelated to HIV, while it was more likely to have been recorded in visits that led to hospital admissions or other facility-based care. Third, despite the significant likelihood of frequent ED visits among patients with HIV and MHSUD,^2^ the effects of MHSUD on return visits by the same individuals could not be examined because observation units were visits, not individuals. Fourth, cross-sectional data allowed estimation of associations but not causality. Future research should investigate both longitudinal and reciprocal effects of MHSUDs on the health status, healthcare utilization, and health outcomes of people with HIV. Fifth, even though the overall number of ED visits by persons with HIV was large, the small sample sizes for combinations of an outcome category and one or more independent variables with low base rates (e.g., discharge AMA by 21–34 years with cognitive disorders) hampered interpretation of the multivariate regression results.

## CONCLUSION

Despite these limitations, the study findings provide significant clinical, research, and policy implications. First, the high prevalence of MHSUDs and their significant roles in ED visit outcomes in patients with HIV provide support for integrated care for these patients^30^ to reduce their ED visits and costly hospital admissions and institutional care that follows. HIV patients’ complex care needs are not likely to be met at EDs unless a holistic care approach that takes into account physical, mental, and social comorbidities (e.g., poverty, lack of consistent access to primary care, and socioeconomic deprivations such as unstable housing^31^) is provided outside the ED. Community resources for psychiatric treatment especially for suicide risk prevention and substance use disorders and case management for unmet needs in other areas should be made available and easily accessible. Second, more research on the relationship between cognitive disorders, other mental disorders, and substance use disorders in patients with HIV and their effects on ED outcomes is needed. Both among younger and older ED patients with HIV, screening and interventions for cognitive disorders are necessary to reduce costly healthcare. Third, in the face of significant shifts in HIV demographics (i.e., graying of HIV patients), systemic efforts to meet the physical and mental health needs of older adults with HIV are necessary. Preventive healthcare and treatment for MHSUD conditions may lead to better HAART/cART adherence and contribute to improving the quality of life for people with HIV while using healthcare dollars more efficiently and effectively.

## Figures and Tables

**Table 1:** Characteristics of patient visits by human immunodeficiency virus (HIV) diagnosis status (%).

Characteristics	No HIV diagnosis (%) 99.51%; N=23,129,163	HIV diagnosis (%) 0.49%; N=115,656
Age group (years)		
21–34	30.02	18.38
35–49	25.16	42.73
50–64	21.71	34.61
65+	23.11	4.29
Gender		
Male	42.67	62.24
Female	57.33	37.76
Primary expected payer		
Medicare	28.89	29.73
Medicaid	19.42	40.06
Private insurance	26.70	11.74
Self-pay	18.85	14.31
Other	5.43	3.36
No charge	0.71	0.80
Median household income in patient’s zip code (national quartile)		
Q1 (<$39,000)	32.86	45.24
Q2 ($39,000–$47,999)	25.53	19.40
Q3 ($48,000–$62,999)	22.53	13.43
Q4 ($63,000+)	17.06	6.70
Missing	2.20	15.23
Diagnosis of physical conditions/diseases		
Infectious and parasitic diseases	8.16	100
Neoplasms	5.37	7.34
Endocrine, nutritional, metabolic and immunity disorders	28.66	36.97
Blood and blood-forming organs	7.63	19.29
Nervous system and sense organs	21.29	25.80
Circulatory system	38.41	47.69
Respiratory system	22.69	35.96
Digestive system	20.73	27.56
Genitourinary system	33.80	42.79
Skin and subcutaneous tissues	6.10	9.75
Musculoskeletal system and connective tissues	20.87	18.21
Other conditions^1^	37.47	46.65
External injury (nonsuicidal)	23.46	15.31
Diagnosis of mental health conditions (MH)		
Anxiety disorders	5.36	5.77
Mood disorders	7.08	15.62
Cognitive disorders	2.63	1.55
Suicide attempt	0.77	2.39
Other mental disorders^2^	2.76	6.20
Diagnosis of substance abuse conditions (SUD)		
Alcohol use disorders (AUD)	3.34	7.45
Drug use disorders (DUD)	2.66	13.75
Co-occurring mental health and substance-use disorders^3^	1.92	7.62

No human immunodeficiency virus (HIV) diagnosis: 99.51% (95% CI [99.51–99.52]). HIV diagnosis: 0.49% (95% CI [0.48–0.49]).

1 Syncope, fever of unknown origin, lymphadenitis, gangrene, shock, nausea and vomiting, abdominal pain, malaise and fatigue, allergic reactions, rehabilitation care, administrative/social admission, and other.

2 Disorders of adjustment, attention deficit, conduct, and disruptive behavior, developmental nature, impulse control, personality, schizophrenia and other psychosis, and other miscellaneous disorders.

3 Any mental disorder and AUD and/or DUD, SE=0.0001 for all visits.

All age group differences are significant at p<0.0001.

**Table 2:** ED outcome by age group and HIV diagnosis status (%).

	Treat-and-release	Hospital admission	Death at ED/hospital	Facility transfer/HHC	Discharge AMA
All adults age 21+^1^					
No HIV diagnosis	77.29	18.87	0.65	1.60	1.72
HIV diagnosis	57.25	38.01	1.06	1.41	2.26
Age 21–34					
No HIV diagnosis	90.02	7.02	0.07	0.87	2.03
HIV diagnosis	66.51	29.13	0.61	1.29	2.45
Age 35–49					
No HIV diagnosis	84.76	11.93	0.19	1.04	2.09
HIV diagnosis	59.21	36.24	0.80	1.45	2.33
Age 50–64					
No HIV diagnosis	74.09	22.14	0.63	1.28	1.87
HIV diagnosis	51.87	43.05	1.48	1.41	2.19
Age 65+					
No HIV diagnosis	55.65	38.75	1.93	2.87	0.80
HIV diagnosis	41.59	53.09	2.30	1.82	1.20

*ED,* emergency department; *HHC,* home healthcare; *AMA,* against medical advice; *HIV,* human immunodeficiency virus

1 N=23,100,490 visits without HIV diagnosis and N=115,578 visits with HIV diagnosis; other/unknown outcomes (0.13% of total without HIV and 0.06% of total with HIV) were excluded from analysis.

Differences by HIV diagnosis status in each age group are significant at p<0.0001.

**Table 3:** Visits by patients with human immunodeficiency virus diagnosis: characteristics by age group (%).

	21–34 years 18.38%; N=21,200	35–49 years 42.73%; N=49,267	50–64 years 34.61%; N=40,122	65+ years 4.29%; N=5,067
Gender				
Male	62.19	59.85	64.47	68.29
Female	37.81	40.15	35.53	31.71
Primary expected payer				
Medicare	14.35	26.48	34.91	86.19
Medicaid	40.68	42.19	41.44	5.20
Private insurance	12.73	11.85	11.87	5.35
Self-pay	27.67	14.99	7.98	1.38
Other	3.53	3.54	3.25	1.73
No charge	-	-	-	-
Median household income in patient’s zip code (national quartile)				
Q1 (<$39,000)	45.52	45.83	44.70	42.47
Q2 ($39,000–$47,999)	21.34	19.94	17.82	18.42
Q3 ($48,000–$62,999)	15.14	13.21	12.95	12.21
Q4 ($63,000+)	6.52	6.62	6.68	8.60
Missing	11.48	14.40	17.86	18.29
Diagnosis of physical conditions/diseases				
Neoplasms	4.11	6.44	9.22	14.88
Endocrine, nutritional, metabolic and immunity disorders	22.03	32.93	46.46	64.66
Blood and blood-forming organs	16.34	18.55	20.89	26.40
Nervous system and sense organs	21.57	26.30	27.16	27.95
Circulatory system	25.42	43.27	60.75	81.63
Respiratory system	30.28	35.34	39.12	41.06
Digestive system	25.60	26.77	28.82	33.68
Genitourinary system	38.63	40.82	45.43	59.00
Skin and subcutaneous tissues	11.39	9.71	9.13	8.17
Musculoskeletal system and connective tissues	12.39	17.67	21.36	22.97
Other conditions^1^	44.96	46.66	47.32	48.48
Diagnosis of mental health conditions (MH)				
Anxiety disorders	5.91	6.10	5.52	3.87
Mood disorders	13.97	17.36	15.00	10.40
Cognitive disorders	0.47	1.02	2.19	6.20
Suicide attempt	2.64	2.91	1.85	0.47
Other mental disorders^2^	6.69	6.66	5.72	3.36
Diagnosis of substance abuse conditions (SUD)				
Alcohol use disorders (AUD)	4.26	8.07	8.86	3.63
Drug use disorders (DUD)	11.38	15.01	14.36	5.44
Co-occurring MHSUD^3^	6.85	8.96	7.16	2.39

21–24 years: 18.38% (95% CI [18.15–18.61]); 35–49 years: 42.73% (95% CI [42.43–43.02]); 50–64 years: 34.61% (95% CI [34.33–34.89]); 65+ years: 4.29% (95% CI [4.17–4.41]).

*MHSUD,* mental health and substance-use disorder

1 Syncope, fever of unknown origin, lymphadenitis, gangrene, shock, nausea and vomiting, abdominal pain, malaise and fatigue, allergic reactions, rehabilitation care, administrative/social admission, and other.

2 Disorders of adjustment, attention deficit, conduct, and disruptive behavior, developmental nature, impulse control, personality, schizophrenia and other psychosis, and other miscellaneous disorders.

3 Any mental disorder and AUD and/or DUD, SE=0.0001 for all visits.

All age group differences are significant at p<0.0001.

**Table 4:** Associations of age group and mental health and substance use disorders with emergency department (ED) outcomes among ED visits by persons with HIV diagnosis: Relative risk ratios (RRR) and 95% confidence intervals (CI) from multinomial logistic regression analysis.

	Treat-and-release vs.			
	
	Hospital admission RRR (95% CI)	Death RRR (95% CI)	Transfer to facility/HHC RRR (95% CI)	Discharge AMA RRR (95% CI)
Age group				
(21–34 years)	-	-	-	-
35–49 years	0.99 (0.95–1.04)	0.97 (0.78–1.20)	1.22 (1.61–1.66)*	1.01 (0.90–1.13)
50–64 years	1.06 (1.01–1.11)*	1.30 (1.04–1.62)*	1.58 (1.34–1.85)‡	1.01 (0.90–1.14)
65+ years	1.27 (1.17–1.30)‡	1.48 (1.10–1.97)†	3.08 (2.36–4.02)‡	0.66 (0.49–0.88)†
Anxiety disorders	1.26 (1.17–1.36)‡	0.35 (0.22–0.57)‡	1.72 (1.44–2.05)‡	1.14 (0.94–1.38)
Mood disorders	1.81 (1.72–1.90)‡	0.70 (0.56–0.88)†	2.73 (2.40–3.12)‡	0.88 (0.76–1.01)
Cognitive disorders	4.52 (3.80–5.37)‡	5.86 (4.20–8.17)‡	6.18 (4.44–8.60)‡	1.31 (0.76–2.26)
Other mental disorders	2.21 (2.05–2.38)‡	1.23 (0.87–1.73)	3.72 (3.20–4.32)	0.94 (0.76–1.15)
Suicide attempt	4.35 (3.84–4.94)‡	0.58 (0.08–4.13)	15.77 (13.05–19.07)‡	0.40 (0.21–0.78)†
Alcohol use disorders	2.00 (1.87–2.14)‡	1.71 (1.33–2.19)‡	1.18 (0.98–1.42)	1.12 (0.94–1.35)
Drug use disorders	4.15 (3.94–4.37)‡	1.57 (1.26–1.96)‡	1.86 (1.60–2.16)‡	1.66 (1.45–1.90)‡

*HHC,* home healthcare; *AMA,* against medical advice; *HIV,* human immunodeficiency virus

Note: The following potential confounders were included but not reported in the table: gender, zip code area median income (lower 50% and missing as opposed to upper 50%), and physical health diagnoses (neoplasms; endocrine, nutritional, metabolic and immunity disorders; blood and blood-forming organs; nervous system and sense organs; circulatory system; respiratory system; digestive system; genitourinary system; skin and subcutaneous tissues; musculoskeletal system and connective tissues; other conditions; and nonsuicidal external injuries).

Model F (96,31090759)=320.55; design df=31,090,854; p<0.0001. N=115,570 visits by all HIV-diagnosed persons aged 21+ years, representing 488,967 weighted ED events.

* p<0.03.

† p<0.01.

‡ p<0.0001.

**Table 5:** Associations of mental health and substance-use disorders with ED outcomes within each age group: Relative risk ratios (RRR) or odds ratios (OR) and 95% confidence intervals (CI) from multinomial or binary logistic regression analysis.

	Treat-and-release vs.			
	
	Hospital admission RRR/OR^1^ (95% CI)	Death RRR (95% CI)	Transfer to facility/HHC RRR (95% CI)	Discharge AMA RRR (95% CI)
Visits by persons 21–34 years old with HIV diagnosis (N=21,067 visits, representing 89,338 weighted ED events)				
Anxiety disorders	1.17 (0.98–1.39)	-	2.11 (1.42–3.15)§	1.17 (0.80–1.71)
Mood disorders	1.91 (1.68–2.17)§	-	3.97 (2.81–5.61)§	1.05 (0.77–1.43)
Cognitive disorders	11.11 (4.60–26.84)§	-	13.44 (2.71–69.10)	#
Other mental disorders	2.44 (2.07–2.89)§	-	3.80 (2.71–5.33)§	0.63 (0.38–1.04)
Suicide attempt	3.56 (2.69–4.70)§	-	12.20 (7.98–18.66)§	0.27 (0.07–1.11)
Alcohol use disorders	2.35 (1.92–2.87)	-	0.79 (0.44–1.42)	1.57 (0.99–2.50)
Drug use disorders	4.40 (3.87–5.00)§	-	1.57 (1.07–2.33)*	1.47 (1.04–2.08)
Visits by persons 35–49 years old with HIV diagnosis (N=49,219 visits, representing 208,874 weighted ED events)				
Anxiety disorders	1.25 (1.11–1.40)§	0.36 (0.17–0.77)†	1.44 (1.10–1.89)†	1.12 (0.84–1.50)
Mood disorders	1.81 (1.69–1.95)§	0.74 (0.52–1.06)	2.91 (2.38–3.56)§	0.87 (0.71–1.07)
Cognitive disorders	5.09 (3.76–6.90)§	7.29 (3.90–13.62)§	5.03 (2.42–10.44)§	0.70 (0.17–2.93)
Other mental disorders	2.51 (2.25–2.80)§	1.08 (0.59–1.98)	3.48 (2.77–4.38)§	0.95 (0.70–1.29)
Suicide attempt	4.44 (3.72–5.30)§	1.65 (0.23–12.03)	16.76 (12.79–21.95)§	0.56 (0.25–1.26)
Alcohol use disorders	2.15 (1.95–2.37)§	2.15 (1.41–3.27)§	1.28 (0.98–1.67)	1.12 (0.86–1.46)
Drug use disorders	4.07 (3.77–4.40)§	1.30 (0.90–1.87)	1.71 (1.37–2.14)§	1.65 (1.35–2.02)§
Visits by persons 50–64 years old with HIV diagnosis (N=40,093 visits, representing 169,228 weighted ED events)				
Anxiety disorders	1.37 (1.20–1.56)§	0.40 (0.20–0.79)†	2.21 (1.66–2.95)§	1.17 (0.83–1.65)
Mood disorders	1.77 (1.62–1.92)§	0.59 (0.40–0.87)†	2.06 (1.65–2.57)§	0.71 (0.54–0.93)*
Cognitive disorders	3.70 (2.87–4.77)§	5.38 (3.39–8.55)§	5.96 (2.87–4.78)§	1.89 (1.03–3.48)*
Other mental disorders	1.90 (1.66–2.17)§	1.32 (0.81–2.15)	3.70 (2.87–4.78)§	1.09 (0.79–1.52)
Suicide attempt	5.64 (4.38–7.26)§	#	14.82 (10.33–2.26)§	0.18 (0.02–1.28)
Alcohol use disorders	1.92 (1.73–2.12)§	1.71 (1.22–2.39)†	1.29 (0.96–1.72)	0.94 (0.70–1.27)
Drug use disorders	4.17 (3.83–4.55)	1.53 (1.12–2.09)†	2.31 (1.82–2.91)†	1.70 (1.35–2.14)§
Visits by persons 65+ years old with HIV diagnosis (N=4.689 visits, representing 19,863 weighted ED events)				
Anxiety disorders	1.30 (0.87–1.95)	-	-	-
Mood disorders	1.44 (1.07–1.95)*	-	-	-
Cognitive disorders	6.09 (4.03–9.22)§	-	-	-
Other mental disorders	1.13 (0.68–1.87)	-	-	-
Suicide attempt	1.77 (0.52–6.01)	-	-	-
Alcohol use disorders	1.93 (1.20–3.10)†	-	-	-
Drug use disorders	2.53 (1.70–3.78)§	-	-	-

*HHC*, home healthcare; *AMA*, against medical advice; *ED*, emergency department; *HIV*, human immunodeficiency virus

1 RRR for the 21–34, 35–49, and 50–64 age groups and OR for the 65+ age group.

Note: In all models, the following potential confounders were included but not reported in the table: gender, zip code area median income (lower 50% and missing as opposed to upper 50%), and physical health diagnoses (neoplasms; endocrine, nutritional, metabolic and immunity disorders; blood and blood-forming organs; nervous system and sense organs; circulatory system; respiratory system; digestive system; genitourinary system; skin and subcutaneous tissues; musculoskeletal system and connective tissues; other conditions; and nonsuicidal external injuries).

# Denotes cases of complete or quasi-complete separation due to low or non-existent combinations of an outcome category and one or more independent variable (e.g., there were no visits by 50–64 year olds who attempted suicide and died). These parameters were not interpreted due to deflated standard errors for which there is no maximum likelihood estimate.

Model F (66,31070953)=144.50, design df=31,071,018, p<0.0001for the 21–34 age group.

Model F (88,31090892)=147.79, design df=31,090,892, p<0.0001 for the 35–49 age group.

Model F (88,31090824)=1155.80, design df=31,090,911, p<0.0001 for the 50–64 age group.

Model F (22,30593748)=50.37, design df=30,593,769, p<0.0001 for the 65+ age group.

* p<0.04.

† p<0.01.

§ p<0.0001.
